# Examining the Obstacles to Timely Otolaryngology Care in Ethiopia and Zimbabwe: A Comparative Analysis

**DOI:** 10.1002/oto2.70078

**Published:** 2025-02-07

**Authors:** Daniel G. Eyassu, Estephania Candelo, Katerina J. Green, Katherine P. Wallerius, Brhanu H. Asgedom, Joshua P. Wiedermann

**Affiliations:** ^1^ Department of Otolaryngology–Head and Neck Surgery Mayo Clinic Rochester Minnesota USA; ^2^ Department of Otolaryngology–Head and Neck Surgery Mayo Clinic Jacksonville Florida USA; ^3^ Centro de Investigaciones Clínicas, Fundación Valle del Lili Cali Colombia; ^4^ Department of Otolaryngology–Head and Neck Surgery Ayder Comprehensive Specialized Hospital Mekelle Ethiopia

**Keywords:** Ethiopia, global surgery, otolaryngology, Three Delays model, Zimbabwe

## Abstract

This study compares delays in otolaryngologic care between patients in Mekelle, Ethiopia and Harare, Zimbabwe using the Three Delays model. Patient surveys conducted among 46 patients in Zimbabwe during October 2022 and 105 patients in Ethiopia during June 2023 revealed a significantly higher prevalence of delays in seeking and reaching care in Ethiopia. This was in the aftermath of the Tigray War, which damaged the region's health care infrastructure and diminished the trust of patients. In Zimbabwe, there was poor awareness among patients and nonotolaryngologist providers of otolaryngology disease and care capacity leading to delays in seeking and reaching care. Patients in both countries faced delays in receiving appropriate care due to resource limitations, with Ethiopia's constraints worsened by the recent war and Zimbabwe's by consistent health care underfunding. The longest delays were observed in head and neck oncology care. These findings provide a foundation for understanding otolaryngologic care delays in these low‐ and middle‐income countries.

The otolaryngologic disease burden is substantial in low‐ and middle‐income countries (LMICs), where people often face challenges in accessing timely care.^1^, ^2^, ^3^, ^4^ The Three‐Delays model, originally developed to mitigate delays leading to maternal mortality, offers a structured approach to understanding care delays.^5^ It identifies 3 types of delays leading to adverse outcomes: delays in deciding to seek care (type 1), reaching care (type 2), and receiving appropriate care (type 3). This model has been widely applied in medicine to identify barriers in timely care^6^, ^7^, ^8^, ^9^ but not in otolaryngology. We conducted studies using this model to investigate the delays faced by patients seeking outpatient otolaryngologic care in Ethiopia and Zimbabwe. This study presents and compares these delays to provide an initial step to understanding otolaryngologic care delays in LMICs.

## Methods

Patient surveys on otolaryngology care delay were conducted at outpatient clinics in Harare, Zimbabwe, and Mekelle, Ethiopia. In Zimbabwe, 46 patients were surveyed during a 4‐week surgical trip in October 2022 at 1 private and 3 public tertiary hospitals. In Ethiopia, 105 patients were surveyed at a public tertiary hospital during a 3‐week trip in June 2023. Semistructured surveys on health care delays were conducted, with responses classified as type 1, 2, or 3 delays based on alignment with the model (Supplemental Tables S1 and S2, available online). Two reviewers independently categorized responses, with discrepancies resolved by a third reviewer. Categorized responses were analyzed in Excel (Microsoft) to summarize delay lengths and prevalence by otolaryngology subspecialty. *P* values for prevalence differences were calculated using *χ* ^2^ tests of independence.

## Results

The delay lengths and common types by patients' age distribution are shown in **Table** 1. In Ethiopia, type 1 delays were reported by 100% of patients (n = 105), type 2 by 75.2% (n = 79), and type 3 by 77.1% (n = 81). In Zimbabwe, type 1 delays were reported by 34.7% of patients (n = 16), type 2 by 36.9% (n = 17), and type 3 by 67.4% (n = 31). The prevalence of type 1 and 2 delays differed significantly between the 2 locations (*P* < .0001) but type 3 delays did not (*P* = .2076). The 3 most common reasons for the delay within type 1, type 2, and type 3 delays are shown in **Table** 2.

**Table 1:** Age Distribution, Average Delay Lengths, and Most Common Type of Delays Among the Patient Samples in Ethiopia and Zimbabwe

Age group, y	Location	Number of patients	Percentage of sample	Average delay length, mo	Most common delay type
0‐2	Ethiopia	4	3.8	5	Type 1
	Zimbabwe	13	28.3	3	Type 3
3‐18	Ethiopia	9	8.6	23	Type 1
	Zimbabwe	16	34.8	6	Type 2 and 3 equally
19‐24	Ethiopia	29	27.6	14	Type 1
	Zimbabwe	2	4.3	1	Type 1 and 2 equally
25‐34	Ethiopia	24	22.9	9	Type 1
	Zimbabwe	2	4.3	0	Type 3
35‐44	Ethiopia	19	18.1	19	Type 1
	Zimbabwe	1	2.1	1	Type 1
45‐54	Ethiopia	9	8.6	32	Type 3
	Zimbabwe	3	6.5	42	Type 1
55‐64	Ethiopia	6	5.7	88	Type 1
	Zimbabwe	4	8.7	7	Type 3
65+	Ethiopia	5	4.8	22	Type 1
	Zimbabwe	5	10.9	5	Type 3

**Table 2:** Description of the 3 Most Common Delays to Otolaryngology Care in Ethiopia and Zimbabwe

Delay	Ethiopia	Zimbabwe
Type 1	Perceived poor quality of care at the initial health care facility (n = 100; 95.2%)	Medical literacy deficiency (n = 9; 19.6%)
	Nearest health care facility inconvenient to visit (distance or inaccessible) (n = 94; 89.5%)	Lack of trust in health care system (n = 4; 8.7%)
	Needed to raise/save funds before seeking care (n = 85; 80.9%)	Sought care from a traditional healer (n = 2; 4.3%)
Type 2	Long travel time and distance to a health care facility (n = 68; 64.8%)	Inadequate referral system from 1 health care facility to a more appropriate one (n = 10; 21.7%)
	Deficient funds for travel costs (n = 31; 29.5%)	Deficient funds for travel costs (n = 7; 15.2%)
	Poor road condition or terrain (n = 24; 22.9%)	Appropriate health care facility not local, requiring distant travel (n = 5; 10.9%)
Type 3	Lack of treatment guidelines (n = 81; 77.1%)	Wrong diagnosis or treatment (n = 19; 41.3%)
	Long wait time before treatment was received (n = 63; 60.0%)	Shortage of equipment and supplies (n = 10; 21.7%)
	Shortage of equipment and supplies (n = 51; 48.6%)	Long wait time before treatment was received (n = 6; 13.0%)

Subspecialty‐specific comparison of delays is shown in **Table** 3. Ethiopian patients had longer average delays than Zimbabwean patients across all subspecialties, except rhinology. Head and neck patients had the longest delays in both settings.

**Table 3:** Most Common Delays and Average Length of Delay by Otolaryngologic Subspecialty

Subspeciality	Location	Number of patients (% of sample)	Most common delay type	Average length of all delays, mo
Laryngology	Ethiopia	9 (8.6)	Type 3	8
	Zimbabwe	9 (19.6)	Type 3	3
Rhinology	Ethiopia	6 (5.7)	Type 1	10
	Zimbabwe	7 (15.2)	Type 2	19
Trauma	Ethiopia	18 (17.1)	Type 1	6
Otology	Ethiopia	48 (45.7)	Type 1	9
	Zimbabwe	19 (41.3)	Type 3	1
Head and neck	Ethiopia	24 (22.8)	Type 3	62
	Zimbabwe	11 (23.9)	All types equally	19

## Discussion

Although Ethiopia and Zimbabwe are both LMICs and share challenges typical of such contexts,^10^, ^11^, ^12^ comparison revealed prevalence and reasons for delays in otolaryngologic care differ. Ethiopian patients reported a significantly higher prevalence of delays in deciding to seek care than Zimbabwean patients, primarily due to poor primary‐level care, transportation barriers, and financial constraints. Surveys in Mekelle were conducted in the aftermath of a 2‐year war that destroyed physical and health care infrastructure in the region.^13^, ^14^ Additionally, many patients presenting to Mekelle were rural residents with a newly developed distrust of government institutions due to the war. These factors combined with the war's economic burden^15^ and safety concerns left people hesitant and fearful to seek care. In Zimbabwe, delays in seeking care primarily stemmed from patients expecting symptoms to self‐resolve or become severe enough to necessitate care. Addressing these problems requires better patient education in otolaryngologic disorders and treatment options and stronger health care infrastructure to build trust.

Delays in reaching care in Ethiopia were predominantly due to transportation and financial constraints exacerbated by the war. In Zimbabwe, the primary reason for delay was poorly organized and misdirected referrals due to providers' insufficient awareness of otolaryngologic disease and care capacity. A study in a similar setting in Zambia found that 67.4% of patients referred to otolaryngologists were misdiagnosed and 50.4% inappropriately referred.^16^ These problems demonstrate the need for improving non‐otolaryngologists' training in otolaryngologic care and addressing inefficiencies of Zimbabwe's referral system.^17^

After reaching health care facilities, Ethiopian and Zimbabwean patients reported not receiving appropriate treatment due to insufficient hospital resources. Lack of appropriate care guidelines and wrong diagnoses and treatments largely reflected poor accessibility of diagnostic and treatment resources.^18^ Although both countries face resource limitations, their reasons are constructs of different socioeconomic contexts. While Ethiopia faces limitations in the wake of a civil war,^13^ Zimbabwe faces a deteriorated health care system due to chronic underfunding and a prolonged economic crisis.^19^

The higher prevalence of otology complaints in both settings suggests a relatively better capacity or greater need for otology care.^20^ Longer delays in Ethiopia across most subspecialties, with the most common delays being type 3 or type 1, indicate greater health care resource constraints and transportation and financial barriers. Head and neck presentations had the longest delays in both settings, with type 3 being the most common. This was primarily due to the scarcity of diagnosis and treatment resources for head and neck cancer. Study limitations include using an unvalidated survey, limited and varying sample sizes, and sampling bias of only patients who reached referral hospitals. Future research should focus on larger samples and validated surveys at lower‐level health care facilities.

In Ethiopia, war‐related distrust and infrastructure destruction exacerbated delays. Zimbabwe's inefficient referral system calls for improved training for nonotolaryngologist providers. Both settings require efforts in patient education, trust‐building, and improved transportation infrastructure. Deficiencies in diagnostic and treatment resources, especially for head and neck cancer, were major contributors to delay. These findings offer initial insights and call for further investigation for future interventions.

## Author Contributions

**Daniel G. Eyassu**, data interpretation, initial manuscript creation, manuscript revision; **Estephania Candelo**, data collection, data interpretation, initial manuscript creation, manuscript revision; **Katerina J. Green**, data collection, data interpretation, initial manuscript creation, manuscript revision; **Katherine P. Wallerius**, data collection, data interpretation, manuscript revision; **Brhanu H. Asgedom**, data collection, data interpretation, manuscript revision; **Joshua P. Wiedermann**, data interpretation, initial manuscript creation, manuscript revision.

## Disclosures

### Competing interests

The authors declare that there are no conflicts of interest.

### Funding source

None.

## Supporting information


**Supplemental Table** 1. Questions for semi‐structured interview of patients and associated delay types. **Supplemental Table** 2. Classification guide for survey responses of delays in otolaryngologic care based on the Three Delays model.
