# Supplementary material for: Examining the Obstacles to Timely Otolaryngology Care in Ethiopia and Zimbabwe: A Comparative Analysis
Source: OTO Open. 2025 Feb 7;9(1):e70078. doi: 10.1002/oto2.70078 (PMC11803452; doi:10.1002/oto2.70078)
Supplement: Supplementary file 1 — Supplemental Table 1. Questions for semi‐structured interview of patients and associated delay types. Supplemental Table 2. Classification guide for survey responses of delays in otolaryngologic care based on the Three Delays model. [file OTO2-9-e70078-s001.docx]

**Supplemental Table 1.** Questions for semi-structured interview of patients and associated delay types.

| **Question** | **Associated Type of Delay** |
| --- | --- |
| How long was it from when your problem started until you sought care? | Type 1 |
| How far do you live from here? How long did it take? How did you get here? | Type 2 |
| Have you tried other treatments for this problem before? Was this advised by another doctor/nurse or a traditional healer? | Type 1, 2 or 3 |
| Did you receive any treatments for your problem that did not work or for an incorrect diagnosis? | Type 3 |
| How many healthcare professionals were you seen by before receiving appropriate treatment for your problem? | Type 2 or 3 |
| Do you feel like there where barriers preventing you from presenting sooner? Why didn’t you come sooner? | Type 1, 2 or 3 |

**Supplemental Table 2.** Classification guide for survey responses of delays in otolaryngologic care based on the Three Delays model.

| **Type 1 (Seeking Care)** | **Type 2 (Reaching Care)** | **Type 3 (Receiving Appropriate Care)** |
| --- | --- | --- |
| Needed to raise/save funds before seeking care | Appropriate healthcare facility not local, requiring distant travel | Long wait time before treatment could be received |
| Delay in realizing that medical care was needed | Had to visit one or more healthcare center(s) who did not have the capacity to treat the issue | Lack of surgical equipment/supplies required for treatment |
| Lack of awareness of treatment options of known medical problem | Reliance on public transportation | Lack of anesthesia equipment/supplies required for treatment |
| Nearest healthcare facility inconvenient to visit (distance or inaccessible) | Deficient funds for travel costs | This facility cannot provide the appropriate treatment due to training deficiencies |
| Previously poor experience/lack of trust of healthcare system | Poor or unsafe road/travel conditions | Wrong diagnosis given previously |
| Avoided due to risk of long stays in hospital | Appropriate healthcare facility was greater than 2 hours away from where they live | Wrong treatment offered/given previously |
| Sought care from a traditional healer before seeking care from a healthcare facility | Inadequate referral system from one healthcare facility to a more appropriate one | Wrong risk assessment of condition communicated previously |
| Perceived poor quality of care at the healthcare facility | Other | Lack of treatment guidelines |
| Previous experience of similar symptoms that resolved without treatment |  | Other |
| Lack of awareness of potential complications |  |  |
| Other |  |  |
